# Gold–Oligonucleotide Nanoconstructs Engineered to Detect Conserved Enteroviral Nucleic Acid Sequences

**DOI:** 10.3390/bios11070238

**Published:** 2021-07-14

**Authors:** Veeren M. Chauhan, Mohamed M. Elsutohy, C. Patrick McClure, William L. Irving, Neil Roddis, Jonathan W. Aylott

**Affiliations:** 1 School of Pharmacy, Boots Science Building, University of Nottingham, Nottingham NG7 2RD, UK; jon.aylott@nottingham.ac.uk; 2 TBG Solutions Ltd. 3A Midland Court, Barlborough Links, Barlborough, Chesterfield, Derbyshire S43 4UL, UK; neil.roddis@manchester.ac.uk; 3 Schulich School of Engineering, University of Calgary, Calgary, AB T2N 4V8, Canada; mohamed.elsutohy@ucalgary.ca; 4 Queen’s Medical Centre, School of Life Sciences, University of Nottingham, Nottingham NG7 2UH, UK; patrick.mcclure@nottingham.ac.uk (C.P.M.); will.irving@nottingham.ac.uk (W.L.I.); 5 Jodrell Bank Observatory, University of Manchester, Macclesfield, Cheshire SK11 9DL, UK

**Keywords:** gold nanoparticles, oligonucleotides, gold–oligonucleotide nanoconstructs, lateral flow assay, enterovirus, virus, nucleic acid sequences, UV–Vis, multiplexing, multimodal, variant detection, spectroscopy, point-of-care

## Abstract

Enteroviruses are ubiquitous mammalian pathogens that can produce mild to life-threatening disease. We developed a multimodal, rapid, accurate and economical point-of-care biosensor that can detect nucleic acid sequences conserved amongst 96% of all known enteroviruses. The biosensor harnesses the physicochemical properties of gold nanoparticles and oligonucleotides to provide colourimetric, spectroscopic and lateral flow-based identification of an exclusive enteroviral nucleic acid sequence (23 bases), which was identified through in silico screening. Oligonucleotides were designed to demonstrate specific complementarity towards the target enteroviral nucleic acid to produce aggregated gold–oligonucleotide nanoconstructs. The conserved target enteroviral nucleic acid sequence (≥1 × 10^−7^ M, ≥1.4 × 10^−14^ g/mL) initiates gold–oligonucleotide nanoconstruct disaggregation and a signal transduction mechanism, producing a colourimetric and spectroscopic blueshift (544 nm (purple) > 524 nm (red)). Furthermore, lateral-flow assays that utilise gold–oligonucleotide nanoconstructs were unaffected by contaminating human genomic DNA, demonstrated rapid detection of conserved target enteroviral nucleic acid sequence (<60 s), and could be interpreted with a bespoke software and hardware electronic interface. We anticipate that our methodology will translate in silico screening of nucleic acid databases to a tangible enteroviral desktop detector, which could be readily translated to related organisms. This will pave the way forward in the clinical evaluation of disease and complement existing strategies to overcome antimicrobial resistance.

## 1. Introduction

Enteroviruses are mammalian pathogens that are transmitted either through the gastrointestinal or respiratory pathways. More than 100 different enteroviruses have been identified, and new pathogenic strains are being discovered because of their high mutation and recombination rates [1].

The enterovirus genus currently consists of 15 species (Enterovirus A-L and Rhinovirus A-C) [1], with potentially new species still being identified [2,3]. These species are associated with mild and serious disease [4], which include the common cold [5] and poliomyelitis [6], respectively. Furthermore, because of symptom similarities, patient pressure, and fear of inaccurate clinical evaluation, enterovirus infections are often evaluated as serious bacterial infections. Bacterial infections, unlike enteroviral infections, can be effectively treated with antibiotics [7]; however, if they are left untreated, they can ultimately become life-threatening [8]. As a result, antibiotics are overprescribed [9] and have contributed to the rise of antimicrobial resistance, which is associated with both long-term medical and economic uncertainty [10]. Therefore, an improved understanding of infectious organisms [11] and their detection [12] could enhance therapeutics that effectively cure and alleviate the symptoms of disease.

At present, conventional enterovirus detection instruments, such as electron [13], light, and fluorescence microscopy [14], and methods, such as polymerase chain reaction (PCR) [15] and microtiter plate-based enzyme-linked immunosorbent assay (ELISA) [16], are not always practical in clinical settings. This is because traditional instruments and methods are often uneconomical, require extensive training to correctly operate, and are time-consuming, such that they do not meet the requirements of a typical doctor–patient consultation (~10 min) [17]. Microtiter-based ELISA has demonstrated breakthroughs in the field through the detection of viruses [18] and has been advanced for clinical settings through the development of lateral flow assays [19], which prove to be economical, simple, and rapid [20,21]. Excellent examples of cases in which ELISA-based lateral flow assays are widely implemented include the ubiquitous pregnancy test [22] and more recently SARS-CoV-2 [23]. However, ELISA, which utilises antibodies to detect analytes of interest, is restricted by its potential to detect small molecular weight targets and variable biosynthesis methods [24]. Consequently, efforts have been directed towards the development of rapid, accurate, and economical point-of-care biosensors that capitalise on innovative detection mechanisms that could be used to stratify individuals presenting with the symptoms of viral infections.

Oligonucleotide biosensors [25], such as aptamer-based biosensors [26], are an example of an emerging technology that is beginning to rival existing infection detection systems [27]. This is due to their economical manufacture, ease of production, high stability, and ability to effectively bind targets with high affinity and specificity [28]. Oligonucleotides have been designed to detect an array of biochemically diverse targets, which include low molecular weight compounds [29], proteins [30], nucleic acids [31], bacteria [32], and eukaryotic cells [33]. Furthermore, a number of innovative signal transduction mechanisms have been developed to detect analytical targets [34], which include quantification of fluorescence emission from quantum dot encoding of oligonucleotide-linked nanostructures [35], electrochemical biosensing using oligonucleotide functionalised platinum nanoparticles [36], and magnetic resonance imaging of oligonucleotide functionalised superparamagnetic iron oxide nanoparticles [37]. Sophisticated methods that combine detection modalities, including fluorescence anisotropy and electrochemical detection, [38] of viral particles with ultra-high sensitivities have also been explored.

Oligonucleotides that utilise the spectroscopic properties of gold nanoparticles [39,40] as they transition from aggregated and disaggregated states as a signal transduction mechanism for colourimetric [41] or lateral flow assays [42], have demonstrated enhanced utility and clinical practicality in detecting biochemical markers, such as adenosine and cocaine [43], thrombin [44], ATP [45], and platelet-derived growth factor receptors [46]. Therefore, oligonucleotides specific to viral biomarkers [47] and gold nanoparticles engineered to change their aggregation state when exposed to a target could be used to stratify patients infected with enteroviruses to improve disease management, augment treatment pathways, and prevent the rise of antimicrobial resistance [48].

In this article, we describe the development of a multimodal gold–oligonucleotide nanoconstruct-based biosensor composed of gold nanoparticles and an in silico designed oligonucleotide sequence that can determine the presence of a target nucleic acid sequence, which is conserved amongst all known enteroviruses, by demonstrating a colourimetric change that can be detected spectroscopically or through the use of lateral flow. Gold nanoparticles were synthesised by sodium citrate reduction. The conserved enteroviral sequence was identified through alignment of archetypal enteroviral nucleic acid sequences, from which a specific and complementary oligonucleotide sequence was engineered. Gold–oligonucleotide nanoconstructs were characterised using dynamic light scattering (DLS), transmission electron microscopy (TEM), and ultra-violet spectrophotometric analyses. Lateral flow assays were fabricated through deposition of a streptavidin band on a membrane that can capture dissociated gold nanoparticles functionalised with biotinylated nucleic acid in the presence of a conserved target enteroviral nucleic acid sequence.

## 2. Materials and Methods

### 2.1. Materials

Gold (III) chloride hydrate, sodium citrate trisodium salt, sodium acetate trihydrate, glacial acetic acid Trizma^®^ acetate, sodium chloride, sodium hydroxide, tris(2-carboxyethyl) phosphine hydrochloride, and phosphate buffered saline, were purchased from Sigma Aldrich (Gillingham, UK). A human genomic DNA control was obtained from BioLine (London, UK). Ultra-pure water (18.2 MΩ.cm) was generated by Elga PureLab Ultra 2 (ULXXXGEM2) and DNase & RNase free water was obtained from Gibco (Life Technologies, Loughborough, UK). Oligonucleotide sequences were obtained from Eurogentec (Liège, Belgium). The STM discovery board (STM32F407G-DISC1), Vero board, Kingbright (L-493GT Green LED), LittelFuse (500 mA), AC/DC power supply, straight pin header (2.54 mm Pitch 3 way, 1 row), LDO regulator (LM2940CT-5.0/NOPB), multilayer ceramic capacitor (KEMET 100 nF), aluminium electrolytic capacitor 100 μF (Panasonic), radial T leaded PCB mount fuse (LittelFuse 500 mA), pitch 50 way 2 row straight PCB socket (Through Hole, Stelvio Kontek MINICOM Series, 2.54 mm), right angle through hole DC power socket (Wurth Elektronik WR-DC series, 5.5 mm, 160 × 90 × 50 mm), trimmer resistor with pin terminations (100 kΩ, Bourns 3296W Series, 25-Turn), turn through hole trimmer resistor with pin terminations (20 kΩ, Vishay 64 W Series 19 (Electrical), 22 (Mechanical)), and ABS enclosure (IP66, 160 × 90 × 60.5 mm) were acquired from RS Components.

### 2.2. Methods

#### 2.2.1. Synthesis of Gold Nanoparticles

Gold chloride (0.017 g) was dissolved in deionised water (0.001 M, 50 mL) and heated under reflux. A solution of sodium citrate tribasic in deionised water (0.039 M, 5 mL) was prepared and added to the reaction mixture with continuous stirring for a further 20 min under reflux. The mixture was cooled to room temperature and stored under refrigeration (2–8 °C).

#### 2.2.2. Synthesis of Gold–Oligonucleotide Nanoconstructs

First, gold nanoparticles were functionalised by linking thiolated nucleic acid to gold nanoparticle surfaces and aggregated through hybridisation of a sensory oligonucleotide strand. The sensory oligonucleotide strand was hybridised by two different complementary nucleic acid strands, such that SH-*Sequence-A* and SH-*Sequence-B-Biotin* were complementary to the 3′ and 5′ ends of the oligonucleotide, respectively. To prepare gold–oligonucleotide nanoconstructs that utilise the signal transduction mechanism of a lateral flow assay, a mixture of SH-*Sequence-A* and SH-*Sequence-B*-Biotin was used in a 1:1 ratio. The biotin-functionalised nucleic acid gold constructs bind membranes doped with streptavidin at predefined locations.

Methods for the formation of gold–oligonucleotide nanoparticle constructs were adapted from a previous study reported by Liu and Lu [49]. Briefly, *Sequence-A* (9 μL, 1 mM) and a mixture of *Sequence-A* (4.5 μL, 1 mM) and *Sequence-B*-Biotin (4.5 μL, 1 mM) were added to two separate sodium hydroxide (12 M)-cleaned vials. Tris(2-carboxyethyl) phosphine (TCEP, 1.5 μL, 10 mM) and acetate buffer (pH 5.2, 1 μL, 500 mM) were added to each vial and incubated at room temperature for 1 hr. Gold nanoparticle suspensions (3 mL) were added to each vial and stirred overnight. Tris acetate buffer (pH 8.2, 30 μL 500 mM) and sodium chloride solution (300 μL, 1 M) were added dropwise to each vial and stirred overnight. Functionalised gold nanoparticle suspensions were transferred to micro-centrifuge tubes and washed with sodium chloride (100 mM)/Tris acetate (25 mM) solution and centrifuged (1 mL, 3 times, 16,110 g, 15 min), discarding the supernatant after each wash. After the final wash, the contents of the vials containing gold nanoparticles conjugated to *Sequence-A* and a mixture of *Sequence-B* and *Sequence-B*-Biotin were combined in a single vial suspended in sodium chloride (300 mM)/Tris acetate (25 mM) buffer solution (1 mL). To this vial, the sensory oligonucleotide strand was added (60 μL, 10 μM) to hybridise with oligonucleotide-functionalised gold and allowed to aggregate overnight under refrigeration (4 °C). The following day the aggregated nanoparticles were washed with sodium chloride (600 mM)/Tris acetate (25 mM) buffer solution and centrifuged (1 mL, 3 times, 800 g, 2 min) to eliminate free sensory oligonucleotide. When the detection limit was 1 × 10^−7^ M and the ratio of target conserved enteroviral nucleic acid to oligonucleotide to *Sequence-A*, *Sequence-B*, and *Sequence-B-Biotin* was 1:1:0.5:0.5, in a 1 mL sample of concentrated gold–oligonucleotide nanoconstructs, the amount of nucleic acid bound to gold nanoparticle surfaces was estimated to be 0.1 nmol for *Sequence-A* and 0.05 nmol for *Sequence-B* and *Sequence-B-Biotin*. The final pellet was suspended in sodium chloride (600 mM)/Tris acetate (25 mM) buffer solution (3 mL) and was ready to use for experimentation or stored under refrigeration (4 °C).

#### 2.2.3. Dynamic Light Scattering (DLS)

Dynamic light scattering was performed using a Malvern Zetasizer Nano ZS. The system was equipped with a 5 mW He-Ne laser source (633 nm), operating at an angle of 173°. Measurements (42 runs, 25 °C) were made using a disposable Sarstedt^®^ polystyrene cuvette. The mean hydrodynamic diameter of the particles was computed from the intensity of the scattered light using Malvern Zetasizer software (6.12). All measurements were conducted in triplicate.

#### 2.2.4. Transmission Electron Microscopy

A suspension of gold nanoparticles was deposited onto a Formvar (3.05 mm) carbon support TEM copper grid and allowed to settle (5 min). Excess nanoparticle suspension was drawn away using filter paper (Whatman, grade 1:11 µm). The particles were imaged using an FEI Tecnai 12 Biotwin TEM (100 kV). All measurements were conducted in triplicate.

#### 2.2.5. Zetasizer

Nanoparticle suspensions were transferred to zetasizer cuvettes (DTS1061, Malvern) and flushed with filtered deionised water. Zeta potential measurements were made in triplicate for nanoparticle suspensions in deionised water and PBS (0.001 M) (parameters used for dispersant deionised water dispersant: refractive index, 1.330; viscosity, 0.8872 cP; dielectric constant, 78.5 εr; Model Smoluchowski F (Ka), 1.5). All samples were equilibrated to 25 °C for 120 s prior to measurement. All measurements were conducted in triplicate.

#### 2.2.6. Ultraviolet Spectrophotometry

Determination of the sensitivity of the biosensor to a conserved target enteroviral nucleic acid sequence was recorded using both changes in absorbance intensity and wavelength shift using a Tecan (Spark 10 M) plate-reading spectrophotometer. The sensitivity was determined by producing log_10_ dilutions of conserved target enteroviral nucleic acid sequence, from 200 μM to 2 pM, followed by the addition of equal volumes of gold–oligonucleotide nanoconstructs to produce a final concentration range from 1 × 10^−3^ M to 1 × 10^−14^ M. All measurements were conducted in triplicate.

#### 2.2.7. Lateral Flow Assay

Lateral flow devices were prepared through assembly of cellulose fibre sample pads (1 cm × 1 cm, Millipore) for solvent absorption and wicking, glass fibre conjugation pads (1 cm × 1 cm, Millipore) for sample deposition, and a HiFlow Plus membrane (1 cm/4 cm, 90 s/4 cm, Millipore) on the surface of adhesive laminated printing paper (see supporting information, Figures S1 and S2). A streptavidin suspension (10 mg/mL) was deposited halfway up the lateral flow membrane to capture disaggregated gold nanoparticles functionalised with *Sequence-B*. A sample of test solution (20 µL) containing gold–oligonucleotide nanoconstructs and a test sample of target enteroviral sequence were deposited on the sample pad of the lateral flow test strips, which were placed in 1 mL of running buffer composed of sodium chloride (600 mM) and tris acetate (25 mM) in deionised water (18.2 MΩ.cm). Lateral flow operation is demonstrated in Supporting Movie S1 (Supplementary Materials).

#### 2.2.8. Cross-Sensitivity/ Selectivity Analyses

To determine whether the gold–oligonucleotide nanoconstructs were able to detect the conserved target enteroviral nucleic acid sequence from simulated mucosal environments, cross-sensitivity analyses were conducted by doping test samples with mass equivalent human genomic DNA (6.15 × 10^−12^ M) prior to biosensor assessment. Cross-sensitivity-analyses were conducted at the detection limit of the conserved target enteroviral nucleic acid sequence of 1 × 10^−7^ M. The efficacy of the gold–oligonucleotide nanoconstructs in the presence of human genomic DNA was conducted in the absence and presence of conserved target enteroviral nucleic acid sequence and assessed via lateral flow assay.

#### 2.2.9. Electronic Desktop Detection

Electronic discrimination of the lateral flow detection band was conducted by monitoring the change in electrical resistance across a light-dependent resistor (LDR) as a function of the amount of light reflected from the surface of the lateral flow membrane. The change in resistance was interpreted on a 12-bit scale (0–4095 au) using a microcontroller (STM discovery) board. The microcontroller board was programmed using Keil (µVision5) so that if resistance fell below or above a predetermined threshold because of a reduction in light reflectance in the presence of a positive detection band, an electronic user interface would read a “POSITIVE” or “NEGATIVE” message. The threshold (2118 au) was set at the output value of the limit of detection for a lateral flow test membrane plus three times the standard deviation (SD) of the noise (2163 au ± 15 SD au). For further information on the assembly of the desktop detector, please see Figure S3 and Supporting Movie S2 (Supplementary Materials).

## 3. Results and Discussion

### 3.1. Design of Viral Sensitive Gold–Oligonucleotide Nanoconstructs

**Figure 1:**
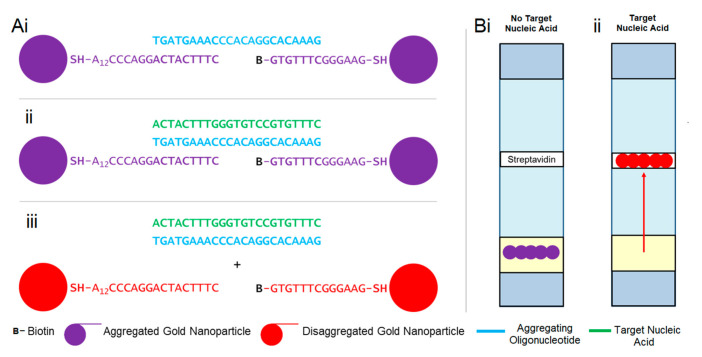
Diagrammatic representation of signal transduction mechanisms of gold–oligonucleotide nanoconstructs using (**A**) colourimetric response and (**B**) differential binding. (Ai) Oligonucleotide 5′ GA AAC ACG GAC ACC CAA AGT AGT 3′ aggregates nanoparticles functionalised with Sequence A (SH-5′ A_12_ CCC AGG ACT AC T TTC 3′) and Sequence B (Biotin—5′ GTG TTT CGG GAA G 3′—SH). (Aii) Target nucleic acid hybridises with oligonucleotide and (Aiii) disaggregates gold nanoparticles to produce a visual and spectroscopic colour change from purple to deep red. (Bi) Aggregated particles do not move upon lateral flow in the absence of target nucleic acid. However, in the presence of (Bii) target nucleic acid, gold particles are disaggregated and able to flow on lateral flow membranes and bind streptavidin because of biotin functionalisation.

Gold–oligonucleotide-nanoconstructs are gold nanoparticles aggregated by oligonucleotides sensitive to a target nucleic acid sequence. When oligonucleotides hybridise with the target nucleic acid sequences, gold nanoparticles disaggregate, initiating a signal transduction pathway (Figure 1), which can be characterised via colourimetric, spectroscopic, and lateral flow assays. Gold–oligonucleotide nanoconstructs sensitive to enteroviral nucleic acids were fabricated via a systematic methodology, which included identification of target nucleic acid and in silico oligonucleotide design, synthesis of gold nanoparticles and functionalisation of their surfaces, and characterisation of the signal transduction pathway.

Sequences for the enterovirus genus were identified using the National Center for Biotechnology Information’s (NCBI’s) “Taxonomy Browser” database. Archetypal representative strains, exclusive to human reference sequences in the enterovirus genus, were selected and aligned using multiple sequence comparison by log-expectation (MUSCLE), which is available in the open-source Molecular Evolutionary Genetics Analysis (MEGA) software. The longest uniformly conserved nucleotide base sequence amongst reference isolates returned was 23 base pairs—5′ ACU UUG GGU GUC CGU GUU UC 3′—which resides in the 5′ non-coding region of the enteroviral genome [50] and therefore will not produce proteins. However, this conserved nucleotide base sequence is in the stem-loop V of the internal ribosome entry site (IRES) [51], binding the single polypyrimidine tract-binding protein. Therefore, depending on sample composition and conditions, a hybridization-based biosensor may be affected by the presence of competing RNA and proteins in real-world settings. The presence of the IRES also suggests that the site is accessible by larger molecular structures, suggesting that there could potentially be an opportunity for the biosensor proposed in this manuscript to detect the conserved nucleotide sequence. For context and the purpose of the experiments detailed in this manuscript, it is also important to note that competing molecules did not affect the performance of the proof-of-concepts studies in this manuscript; however, competing human DNA will be evaluated as part of cross-sensitivity/selectivity analysis in Section 3.4.

NCBI’s basic local alignment search tool (BLAST) highlighted that this sequence was exclusive to the enteroviral genus species and had 100% identity matches with 8291/8648 (96%) sequences. This sequence has also been highlighted in the literature for its utility in PCR priming at a serotypic level [52,53], but not at a universal genus level. Searches of human genomic databases highlighted that the longest sequence matching human DNA was 17 bases on chromosome 12 (GRCh38.p13, 17/23 bases, 74% sequence identity) [54]. This could potentially be a limitation of the biosensor and could produce false positive results; therefore, cross-sensitivity analyses were conducted. Nevertheless, these 23 bases were selected as a target from which a bespoke oligonucleotide sequence was designed.

For the signal transduction pathway to function in the absence of the target nucleic acid sequences, oligonucleotides should aggregate gold nanoparticles. However, in their presence, oligonucleotides must hybridise with target nucleic acid sequences and dissociate from gold nanoparticles, which in turn triggers their disaggregation. This was achieved by designing an oligonucleotide that demonstrates a strong affinity for the target nucleic acid sequence, whilst demonstrating partial affinity for nucleotides linked to the surface of gold nanoparticles, which were used to facilitate their aggregation. Therefore, nucleotide bases specific and complementary to the target nucleic acid were selected for the oligonucleotide sequence 5′ GA AAC ACG GAC ACC CAA AGT AGT 3′ (Figure 1(Ai)).

This oligonucleotide was used to bridge and aggregate gold nanoparticles by partially hybridising two different nucleotide sequences, *Sequence-A* (SH-5′ A_12_ CCC AGG ACT AC T TTC 3′) and *Sequence-B* (Biotin-5′ GTG TTT CGG GAA G 3′-SH), which were linked to gold nanoparticle surfaces via stable thiol-gold bonds [55] (Figure 1(Aii)). *Sequence-A* consisted of thiol functionalisation at 5′ to bind gold nanoparticle surfaces, a polyadenylated tail (*n* = 12) to minimise the interaction with signal transduction components, and 9 nucleotide bases complementary to the 3′ oligonucleotide strand. By contrast, *Sequence-B* was composed of thiol functionalisation at 3′ to bind to gold nanoparticle surfaces and 7 nucleotide bases complementary to the 5′ oligonucleotide strand. The lateral flow device was engineered by biotinylating *Sequence-B* at 5′ (Figure 1(Aiii)) such that it could be trapped by streptavidin-functionalised membranes (Figure 1(Bi,Bii)).

Assembly of gold–oligonucleotide nanoconstructs via this partial nucleic acid complementation method consigns 7 nucleotide bases (5′ G GAC ACC 3′) at the centre of the oligonucleotide as unbound. This ensures that the oligonucleotide will preferentially hybridise with the conserved target enteroviral nucleic acid sequence, thus allowing gold nanoparticles to disaggregate and initiate the signal transduction pathway (Figure 1(Aii,Aiii)).

### 3.2. Synthesis and Characterisation of Gold–Oligonucleotide Nanoconstructs Sensitive to Conserved Target Enteroviral Nucleic Acid Sequences

The spectroscopic (colour and ultraviolet UV absorbance maxima) and physicochemical properties (mobility across lateral flow membranes) of gold nanoparticles differ when they are in disaggregated or aggregated constructs. Therefore, prior to harnessing these properties to develop a biosensor to detect conserved target enteroviral nucleic acid sequences, gold nanoparticles and gold–oligonucleotide nanoconstructs were synthesised, functionalised with *Sequence-A*, *Sequence-B* and oligonucleotide, and characterised for their spectroscopic properties, size, and zeta potential.

Colloidally stable gold nanoparticles were synthesised via sodium citrate reduction. Disaggregated gold nanoparticles exhibit a characteristic deep red colour with a UV absorption maximum at 524 nm (Figure S4). Aggregation of citrate-stabilised gold nanoparticles with a concentrated salt solution red-shifts the wavelength of their absorbance maxima to 544 nm and their appearance to a dark purple colour (Figure S4). Functionalisation of gold nanoparticle surfaces with *Sequence-A* and *Sequence-B* was performed by independently reducing the thiol groups on the nucleic acid sequences in the presence of gold nanoparticle suspensions. Gold–oligonucleotide nanoconstructs were assembled by incubating gold nanoparticles functionalised with *Sequence-A* and *Sequence-B* in a 1:1 ratio, with an initial molar excess of the oligonucleotide strand, overnight. Unbound oligonucleotides and disaggregated gold nanoparticles were removed with washing and centrifugation to produce gold–oligonucleotide nanoconstructs aggregated suspended in a buffer solution composed of sodium chloride (600 mM) and tris acetate (25 mM).

**Figure 2:**
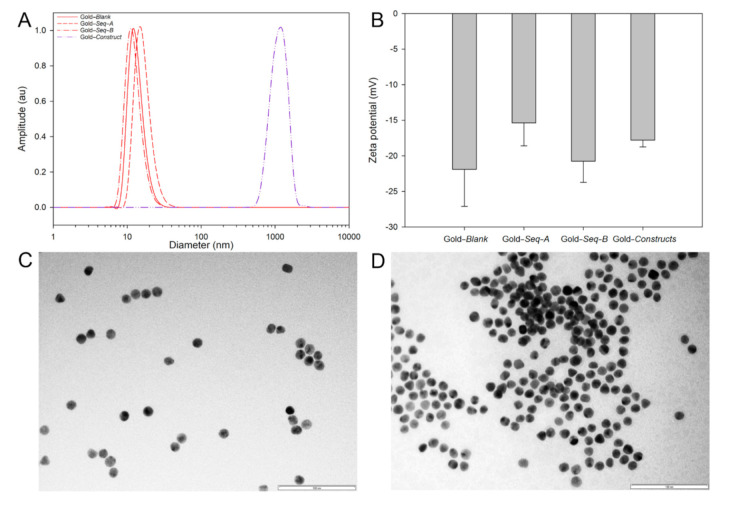
(**A**) Dynamic light scattering-measured mean-centred particle size distribution and (**B**) zeta potential measurements for unfunctionalised gold nanoparticles (Gold–*Blank*), gold nanoparticles functionalised with Sequence–A (Gold–*Seq–A*) and Sequence-B (Gold–*Seq–B*), and gold–oligonucleotide nanoconstructs (Gold–*Constructs*). TEM images for (**C**) unfunctionalised gold nanoparticles and (**D**) gold–oligonucleotide nanoconstructs. Scale bar = 100 nm.

DLS was used to record the mean-centred hydrodynamic diameters for unfunctionalised gold nanoparticles (13 nm diameter, 0.50 PDI), *Sequence-A-* (17 nm diameter, 0.12 PDI) and *Sequence-B* (12 nm diameter, 0.14 PDI)-functionalised gold nanoparticles, and gold–oligonucleotide nanoconstructs (1154 nm diameter, 0.36 PDI) (Figure 2A). Unfunctionalised nanoparticles and *Sequence-A-* and *Sequence-B-*functionalised gold nanoparticles exhibited comparable particle size distributions. The subtle changes in mean hydrodynamic diameter observed by DLS could be attributed to differences in gold nanoparticle bound nucleic acid chain composition, length, and corresponding ionisation (Figure 1, *Sequence-A* 8442.7 Mw, *Sequence-B* 4241.9 Mw, and *Sequence-B-Biotin* 4811.5 Mw). However, when *Sequence-A-* and *Sequence-B*-functionalised gold nanoparticles were aggregated with the sense oligonucleotide, the mean-centred hydrodynamic diameter, as measured with DLS, was considerably larger (50×), which, alongside colourimetric and spectroscopic characterisation, confirmed that gold–oligonucleotide nanoconstructs were fabricated. The relatively large particle size distribution associated with gold–oligonucleotide nanoconstructs suggests that there is heterogeneity during aggregate production, such that their hydrodynamic architecture can span greater than 1 µm in diameter.

The binding of thiolated nucleotide sequences to the surface of gold nanoparticles was confirmed by determining their zeta potential (Figure 2B). Unfunctionalised gold nanoparticles, *Sequence-A-* and *Sequence-B*-functionalised gold nanoparticles, and aggregated nanoparticles exhibited zeta potentials of −21.9 ± 5.21 mV, −15.37 ± 3.23 mV and −20.77 ± 2.95 mV, and −17.8 ± 0.95 mV, respectively. These results suggest that functionalised nanoparticle surfaces exhibit a marginal increase in zeta potential, although not significant (*p* > 0.01), when compared with unfunctionalised nanoparticles. Furthermore, the aggregates, which are formed from a construct of *Sequence-A-* and *Sequence-B*-functionalised gold nanoparticles, as expected, exhibited a zeta potential between the zeta potentials of *Sequence-A-* and *Sequence-B*-functionalised gold nanoparticles.

TEM was used to characterise the size of unfunctionalised and functionalised gold nanoparticles, as well as to determine the distribution of disaggregated unfunctionalised gold nanoparticles and gold–oligonucleotide nanoconstructs. The diameter of unfunctionalized nanoparticles was centred at 13 ± 1.71 nm (*n* = 50, Figure 2C and Figure S5). The size of unfunctionalised and gold nanoparticles functionalised with *Sequence-A* and *Sequence-B* was indistinguishable when imaged using TEM (Figure 2 and Figure S6). This observation could be due to the apparent sizes of *Sequence-A* and *Sequence-B* and the resolution power of the TEM. For example, nucleic acid sequences that are 10 base pairs long are approximately 34 Å (0.34 nm) in length. Therefore, gold nanoparticles functionalised with *Sequence-A* (27 nucleotide bases, 0.918 nm) and *Sequence-B* (13 nucleotide bases, 0.44 nm) would be extremely challenging to differentiate, as the size of the nucleotide sequences falls within the error for unfunctionalised nanoparticles size measurement.

Unfunctionalised gold nanoparticles, when imaged using TEM, were dispersed, appearing as individual nanoparticles or in small clusters (<10 gold nanoparticles) (Figure 2C). In comparison, gold–oligonucleotide nanoconstructs were composed of large nanoparticle complexes (>50 gold nanoparticles) (Figure 2D). The distribution of unfunctionalised gold nanoparticles and gold–oligonucleotide nanoconstructs and the interaction of light with the surface plasmon on free and aggregated nanoparticles could be attributed to the observed visual and spectral differences [56]. Dispersed gold nanoparticles absorb and reflect light at ~500 nm and ~700 nm, respectively, producing a deep red colour. By contrast, gold–oligonucleotide nanoconstructs behave similarly to larger gold nanoparticles, and the surface plasmon resonance absorption wavelength is shifted, such that red light is absorbed, and blue light is reflected, producing a purple colour. These observed visual and spectral differences between disaggregated nanoparticles and gold–oligonucleotide nanoconstructs were used as a signal transduction mechanism to detect conserved target enteroviral nucleic acid sequences.

### 3.3. Detecting Conserved Target Enteroviral Nucleic Acid Sequences with Gold–Oligonucleotide Nanoconstructs

The sensitivity of gold–oligonucleotide nanoconstructs was assessed by challenging the assay to conserved enteroviral sequence containing thymine (or 5-methyuracil, T) (5′ACT ACT TTG GGT GTC CGT GTT TC 3′). Uracil (U) and 5-methyuracil are nucleotide bases that are found in RNA and DNA, respectively, and form hydrogen-bonded base pairs with adenine (A). The A–U hydrogen bond is stronger than the A–T hydrogen bond [57], therefore our reported sensitives are likely to underestimate the ultimate limit of detection. The signal transduction mechanism was investigated using (1) spectroscopy, through observation of the changes in absorbance intensity and the shift in absorbance maxima; (2) visually inspecting the colour changes of aggregates; and (3) application of lateral flow assays.

**Figure 3:**
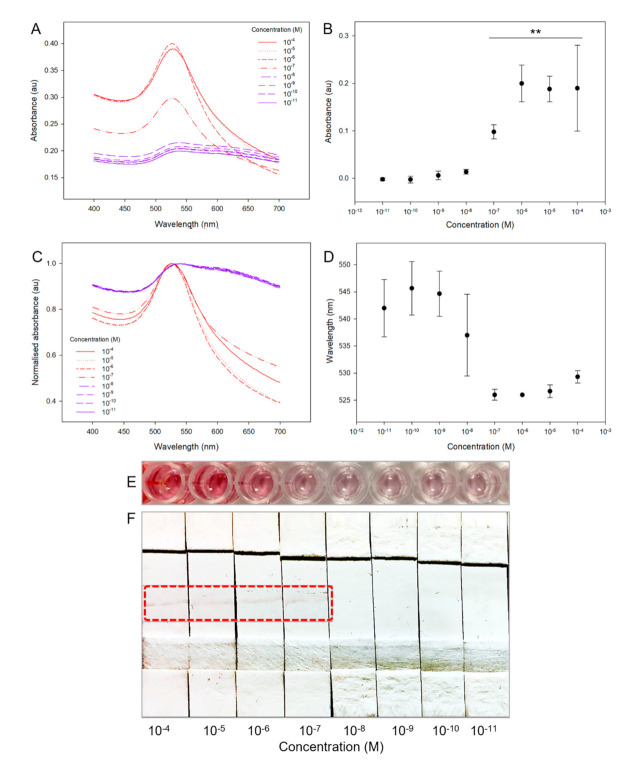
Response of gold–oligonucleotide nanoconstructs to conserved target enteroviral nucleic acid sequence, with concentrations ranging from 1 × 10^−4^ M to 1 × 10^−11^ M, recorded as changes in (**A**,**B**) absorbance intensity, (**C**,**D**) wavelength redshift, (**E**) optical colour change, and (**F**) lateral flow assay, with region of interest highlighted with red dotted line, indicating detection of target nucleic acid sequence. Please note that the light band positioned at the centre of the lateral flow device indicates location of streptavidin deposition. Calibration curves show changes in (**B**) absorbance maxima with subtraction of background absorbance, and (**D**) wavelength (*n* = 3, where error is standard deviation and ** *p* < 0.01).

Spectroscopic analysis of the absorbance response of the gold–oligonucleotide nanoconstructs to log dilutions of target enteroviral nucleic acid, from 1 × 10^−4^ M to 1 × 10^−11^ M, showed that there was a decrease in absorbance amplitude and a blue shift in the absorbance wavelength maxima (Figure 3A). The response of the gold–oligonucleotide nanoconstructs was sigmoidal, without an apparent maximum, because of the finite number of oligonucleotide-based sensing elements that lead to initiation of a signal transduction cascade in the presence of conserved target enteroviral nucleic acid sequences. The absorption maxima intensity of gold–oligonucleotide nanoconstructs at 524 nm were significantly increased in the presence of target enteroviral nucleic acids ≥1 × 10^−7^ M (≥6.02 × 10^−13^ copies/mL, ≥13 log_10_ copies/mL, ≥1.4 × 10^−14^ g/mL, *p* < 0.01; Figure 3B). A detection limit of ≥1 × 10^−7^ M is equivalent to >6.0210^16^ viral particles, when Avogadro’s constant is 6.02 × 10^2^^3^ mol^−1^, and estimating that each viral particle consists of a single copy of the target nucleic acid sequence. It is important to note the data represented in Figure 3B are a true representation of the real-world use of gold–oligonucleotide nanoconstructs. Gold–oligonucleotide nanoconstructs consist of delicate structures that vary from batch to batch and exhibit an absorbance difference. Furthermore, this does not significantly affect their ability to make measurements of target enteroviral nucleic acid ≥1 × 10^−7^ M (*p* < 0.01). These findings were mirrored for the wavelength shift of gold–oligonucleotide nanoconstructs to changes in target enteroviral nucleic acid (Figure 3C), such that shifts in peak absorbance wavelength were also observed at ≥1 × 10^−7^ M (Figure 3D).

Spectroscopic shifts in wavelength of gold–oligonucleotide nanoconstructs to the conserved target enteroviral nucleic acid were also visually observed for conserved target enteroviral nucleic acid concentrations ≥1 × 10^−6^ M, which is above the detection limit of spectroscopy (≥1 × 10^−7^ M). This difference can be attributed to both the enhanced sensitivity of spectroscopy as a detection technique and the limitations of the human eye to determine subtle changes in absorbance and the submaximal spectroscopic response in absorbance and wavelength shift at ≥1 × 10^−7^ M [58]. Therefore, to produce an economical detection technique that is independent of spectroscopy but demonstrates comparable sensitivity, lateral flow assays were investigated.

When gold–oligonucleotide nanoconstructs disaggregate in the presence of the target nucleic acid, they exhibit enhanced flow properties on lateral flow membranes because of a reduction in their physical size (Figure 2C,D). Therefore, free gold nanoparticles functionalised with *Sequence-B*, which contain biotin, were shown to bind streptavidin-doped lateral flow membranes at predetermined sites via one of the strongest non-covalent interactions found in nature [59]. The lateral flow device was able to detect levels of the target viral nucleic acid of ≥1 × 10^−7^ M (Figure 3F). This sensitivity is comparable to the detection limit of the spectroscopic measurement and outperforms visual inspection of the colour change alone. The observed detection limits for the gold–oligonucleotide nanoconstructs were comparable to previously reported gold nanoparticle-based detection systems that have been used for laboratory identification of viruses [60]. Clinically, enteroviral loads are typically acquired from nasal swabs and their concentrations are recorded as log_10_ copies/mL using PCR [61]. Therefore, the detection limit of gold–oligonucleotide nanoconstructs (13 log_10_ copies/mL) would fall outside of the nasal swab based clinically recorded relevant range (5.3 (±1.5)–6.4 (±1.3) log_10_ copies/mL) [62]. It is important to note that our detection limit was determined without any amplification of viral nucleic acid. The difference in detection limit could be overcome through alternate sample collection methods (throat swabs) as well as concentrating viral load through method optimisation, such as the addition of virus isolation and purification steps.

### 3.4. Cross-Sensitivity/Selectivity Analyses

It is anticipated that non-invasive throat swabs, which contain a high number of viral particles during an infection [63], will be utilised when stratifying individuals that are positive or negative for target enteroviral nucleic acid using gold–oligonucleotide nanoconstructs. Therefore, to determine the applicability of the gold–oligonucleotide nanoconstructs in the real-world scenario of a typical doctor–patient consultation, preliminary experiments were conducted to determine the lateral flow assay efficacy at its detection limit (~1 × 10^−7^ M) with equivalent mass concentrations of human genomic DNA (~3.2 g/L, ~6.15 × 10^−12^ M). It is important to note that the molar concentration of human genomic DNA in this experiment was ~10,000× less than that of the target enteroviral nucleic acid, as viral infection of a cell can produce up to 10,000 new viral particles [64]. In the absence of conserved target enteroviral nucleic acid, the assay was negative since human genomic DNA does not initiate the signal transduction pathway (Figure S7). Furthermore, in the presence of conserved target enteroviral nucleic acid, the human genomic DNA did not affect the sensitivity of the lateral flow assay.

The time to detection of the lateral flow assay was also evaluated to determine its utility in clinical settings. This was conducted by monitoring the duration of time taken for the lateral flow assay to produce a detection band at a predetermined streptavidin region of interest. Using our experimental setup, a detection band was observed in <60 s (Supporting Movie S1 (Supplementary Materials)). 

**Figure 4:**
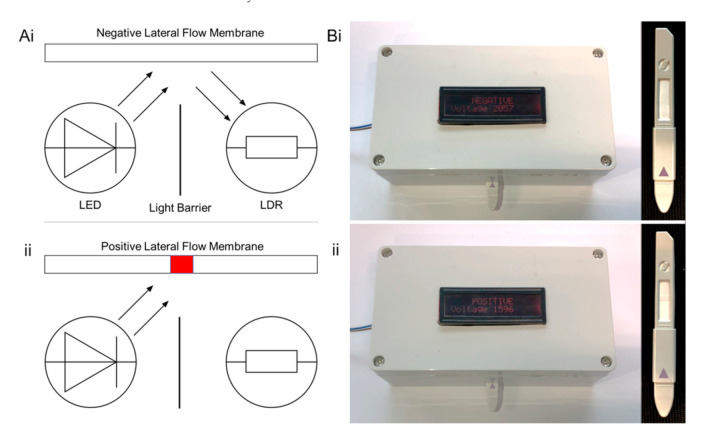
(**A**) Diagrammatic representation and (**B**) photographs of desktop detector response to (Ai,Bi) negative and (Aii,Bii) positive conserved target enteroviral nucleic acid sequence lateral flow assay. For further information see Figure S3 and Supporting Movie S2 (Supplementary Materials).

Furthermore, to establish an unambiguous interpretation of the gold–oligonucleotide nanoconstruct-based lateral flow assay, which could be used by both healthcare professionals and patients, a bespoke software and hardware solution was engineered. The desktop detector was composed of a light-emitting diode (LED) and LDR, positioned perpendicular to a printed circuit board and separated by an opaque light barrier (Figure 4). The light barrier only permits reflected light to be sensed by the LDR; therefore, in the presence of an enteroviral positive lateral flow membrane (Figure 4(Ai)), compared with an enteroviral negative membrane (Figure 4(Aii)), relatively less light was reflected and consequentially sensed by the LDR. The difference in light reflectance corresponded to a change in electrical resistance. A central processing unit was programmed to threshold the change electrical resistance, such that a user interface instantly displayed “NEGATIVE” (Figure 4(Bi)) or “POSITIVE” (Figure 4(Bii)) in the presence or absence of the conserved enteroviral nucleic acid (Supporting Movie S2). Based on our understanding of sample acquisition time (<1 min), preparation time (<2 min), and implementation of the lateral flow assay and desktop detector (<1 min) we anticipate that the total time required to complete an analysis would be ~5 min. This is well within the time frame of a typical doctor–patient consultation (~10 min), providing sufficient time for a thorough clinical evaluation [65] prior to the implementation of a gold–oligonucleotide nanoconstruct enteroviral assay.

## 4. Conclusions

Gold–oligonucleotide nanoconstructs sensitive to a target enteroviral nucleic acid sequence conserved amongst 96% of all known enteroviruses were fabricated. Oligonucleotides were designed in silico to fully complement the target enteroviral nucleic acid and partially hybridise with gold nanoparticles functionalised with complementary nucleic acid. In silico design provides a flexible platform that can be readily optimised to produce multiplexed devices capable of simultaneously detecting an array of analytes. For example, gold–oligonucleotide nanoconstructs could be multiplexed to simultaneously stratify patients presenting with symptoms of viral, bacterial, and mixed viral/ bacterial infections. Furthermore, in the context of the COVID-19 pandemic [66], gold–oligonucleotide nanoconstructs could also be multiplexed to detect SARS-CoV-2 variants [67] or additional viruses (e.g., influenza), which could be used to inform effective use of preventative medicines [68] or treatment pathways [69].

In the presence of the conserved target enteroviral nucleic acid sequence, a signal transduction mechanism was initiated, and gold–oligonucleotide nanoconstructs disaggregated. Multimodal colourimetric (purple to red), spectroscopic (544 nm to 524 nm), and lateral flow assays (binding of biotin functionalised gold nanoparticles to streptavidin-doped membranes) were shown to be sensitive towards the target nucleic acid sequence ≥1 × 10^−7^ M (≥1.4 × 10^−14^ g/mL). This multimodal approach could be used as a powerful control to triangulate the detection of a target nucleic acid sequence between methodologies and provide a summative method for sample negative/positive identification. Future studies will develop negative controls for the lateral flow assay, which could serve as an independent tool for the confirmation or absence of the target nucleic acid sequence.

Lateral flow assays were unaffected by contaminating human DNA, and a complete analysis from sample acquisition to detection could potentially take <5 min. It is also important to note that the reagents, including oligonucleotides, and the electronic components used to prepare the gold–oligonucleotide nanoconstructs and the desktop detector are widely available. Therefore, if mass-produced in comparable economies of scale to the antibody-based pregnancy test, we hypothesise that a fully functioning biosensor could potentially be manufactured for ~10 USD, where the lateral flow device and desktop diagnostics could be produced for <1 USD and <10 USD, respectively.

The research demonstrated in this article indicates that these detection modalities are extremely practical; however, at present lack the required sensitivity to be clinically relevant. The detection sensitivities could be considerably enhanced through the implementation of improved oligonucleotides demonstrating greater specificity for enterovirus structures, such as those for the viral capsid [38]. We anticipate our economical, simple, and rapid assay could be translated into an accessible point-of-care desktop detector device through optimisation of sample preparation, improvements in the detection limit, and evaluation with clinically relevant samples, which include enterovirus, influenza and coronavirus, and other infectious agents. Ultimately, our approach could pave the way forward in the simultaneous detection of multiple viruses and complement existing strategies aimed at overcoming antimicrobial resistance.

## Data Availability

The data presented in this study are available on request from the corresponding author. The data are not publicly available due to data confidentiality.

## Supplementary Materials

The following are available online at https://www.mdpi.com/article/10.3390/bios11070238/s1, Additional content includes Supporting Information PDF. Figure S1. Lateral flow device (A) diagrammatic and (B) actual device representation. Figure S2. Production of lateral flow device using (A) absorption pads, sample pads, lateral flow membranes, and (B) laminated backing paper. Lateral flow devices produced (C) individually and (D) in a high-throughput batch format. Scale bars = 5 cm. Figure S3. (A) Top and (B) cross-sectional view of LED, Light Barrier, and LDR positioning. (C) Internal specification (STM board, LCD display, light-emitting diode (LED) and LDR) and (D) external profile (desktop diagnostic (with later flow cartridge insert), positive (left) and negative cartridges (right) and power outlet). See Supporting Movie S2 for desktop diagnostic operation. Figure S4. Absorbance spectra for disaggregated and aggregated gold nanoparticles. Absorbance maxima for free and aggregated nanoparticles were 524 nm and 544 nm, respectively. Inset image of disaggregated (left) and aggregated (right) gold nanoparticle suspensions. Figure S5. Histogram of unfunctionalized gold nanoparticle size distribution determined from TEM (n = 50). Figure S6. TEM images for gold nanoparticles functionalised with (A) Sequence-A, (B) Sequence-B, and (C) after signal transduction pathway has been initiated following addition of conserved target enteroviral nucleic acid sequence. Scale bars = 100 nm. Figure S7. Response of gold–oligonucleotide nanoconstructs to target viral DNA in the presence of human genomic DNA (0.1 μM), with (A) colourimetric change and (B) lateral flow assay. (C) Summary of lateral flow findings showing that signal transduction mechanism of gold–oligonucleotide nanoconstructs is not initiated in the presence of human genomic DNA (H-DNA, 0.1 μM) and does not affect the triggering of signal transduction cascade for the conserved target enteroviral nucleic acid (CTENA, 0.1 μM). Supporting Movie S1. Rapid colourimetric response of gold–oligonucleotide nanoconstructs in suspension when challenged with conserved target enteroviral nucleic acid (0.1 μM) and the binding of disaggregated biotin-functionalised gold nanoparticles to streptavidin of lateral flow assays. Supporting Movie S2. Differentiation of conserved target enteroviral nucleic acid positive and negative lateral flow cartridges using custom-designed diagnostic.
