# Supplementary material for: Gold–Oligonucleotide Nanoconstructs Engineered to Detect Conserved Enteroviral Nucleic Acid Sequences
Source: Biosensors (Basel). 2021 Jul 14;11(7):238. doi: 10.3390/bios11070238 (PMC8301919; doi:10.3390/bios11070238)
Supplement: Supplementary file 1 [file biosensors-11-00238-s001.zip › biosensors-1272992-sup/MDPI - Supporting Information.pdf]

SUPPORTING INFORMATION

Gold-Oligonucleotide-Nanoconstructs Engineered to Detect Conserved Enteroviral Nucleic Acid Sequences

Veeren M Chauhan [a, b], Mohamed M Elsutohy [a, c], C Patrick McClure [d], William L Irving [d], Neil Roddis [b] and Jonathan W Aylott [a*]*

[a] School of Pharmacy, Boots Science Building, University of Nottingham, Nottingham, *NG7 2RD, United Kingdom*
[b] TBG Solutions Ltd. 3A Midland Court, Barlborough Links, Barlborough, Chesterfield, Derbyshire, S43 4UL, United Kingdom
[c] Schulich School of Engineering, University of Calgary, Calgary, AB T2N 4V8, Canada
[d] School of Life Sciences, Queen's Medical Centre, University of Nottingham, Nottingham, NG7 2UH, United Kingdom

Corresponding authors *
Dr Veeren M Chauhan - Veeren.Chauhan@nottingham.ac.uk
Dr Jonathan W Aylott - Jon.Aylott@nottingham.ac.uk

CONTENTS

Supporting Figures S1 – S7

Captions for Supporting Movies 1-2

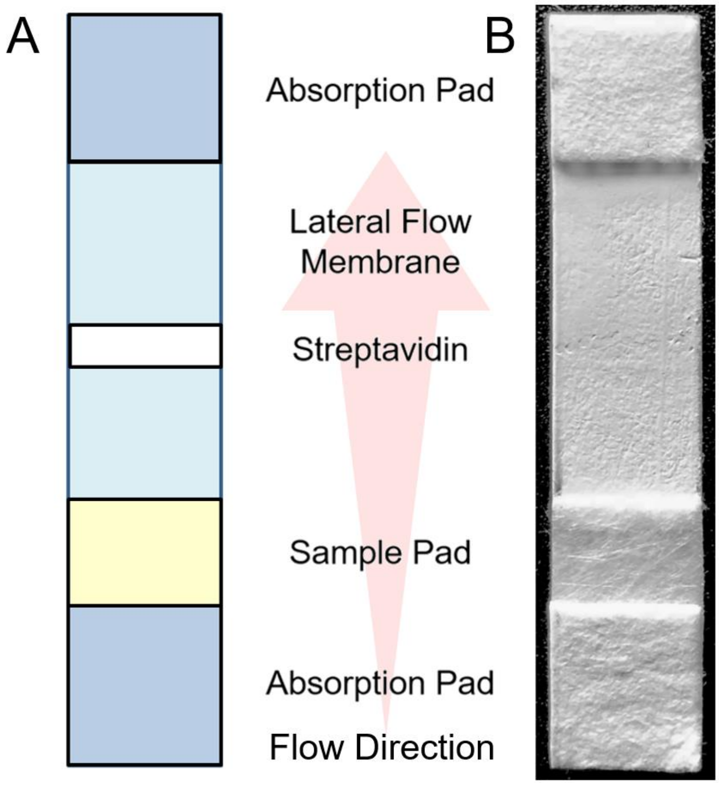

Figure S1. Lateral flow device (A) diagrammatic and (B) actual device representation.

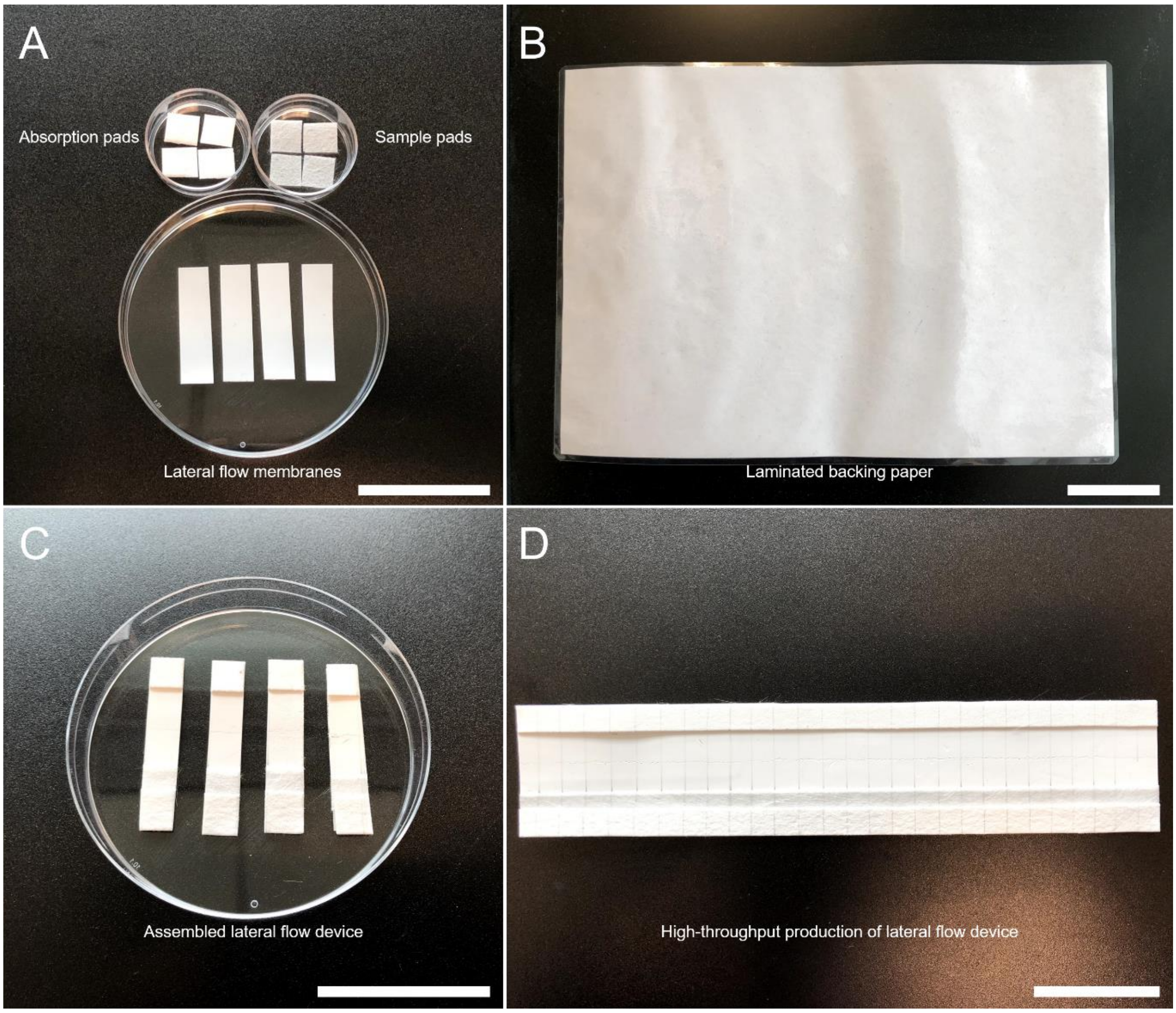

Figure S2. Production of lateral flow device using (A) absorption pads, sample pads, lateral flow membranes and (B) laminated backing paper. Lateral flow devices produced (C) individually and (D) in a high-throughput batch format. Scale bars = 5 cm.

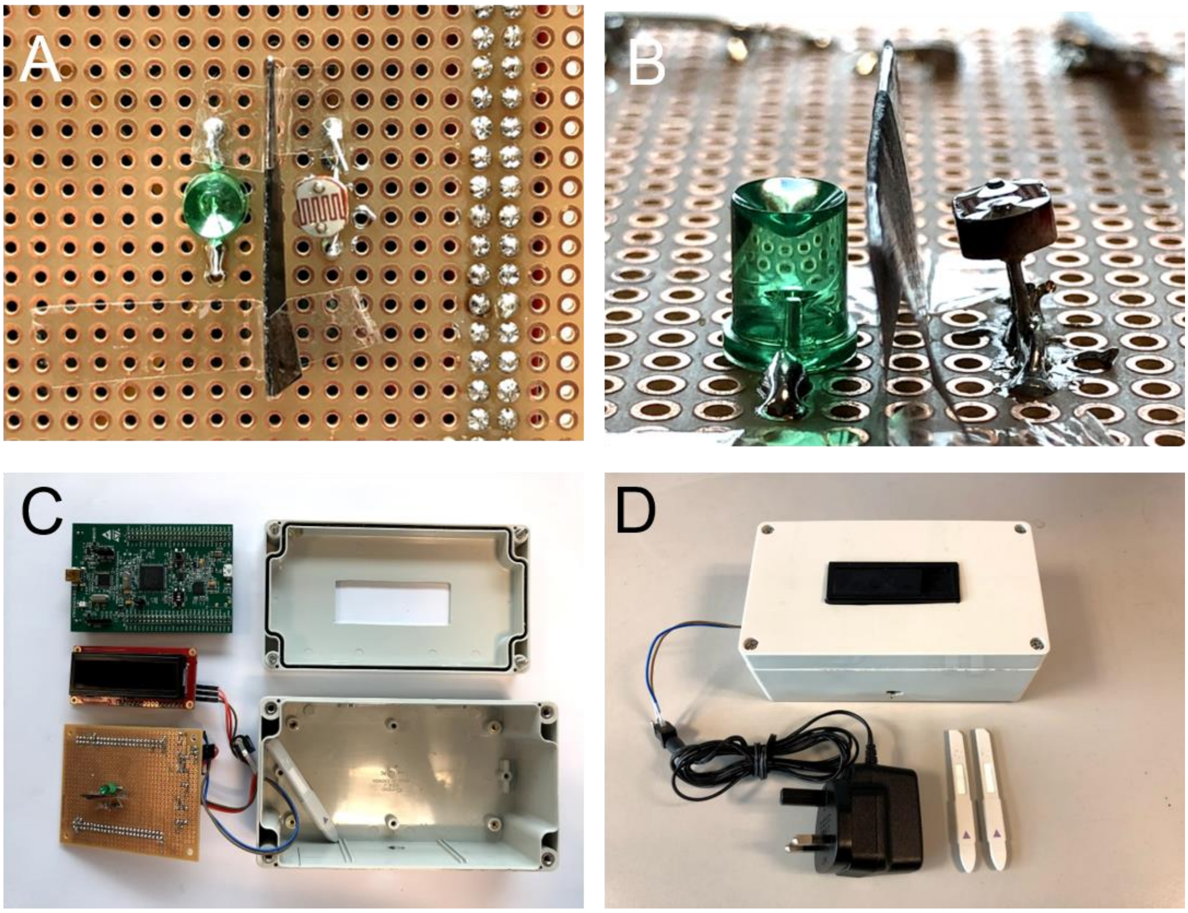

Figure S3. (A) Top and (B) cross-sectional view of LED, Light Barrier and LDR positioning. (C) Internal specification (STM board, LCD display, light emitting diode (LED) & LDR) and (D) external profile (desktop diagnostic (with later flow cartridge insert), positive (left) and negative cartridges (right) and power outlet). See Supporting Movie 2 for desktop diagnostic operation.

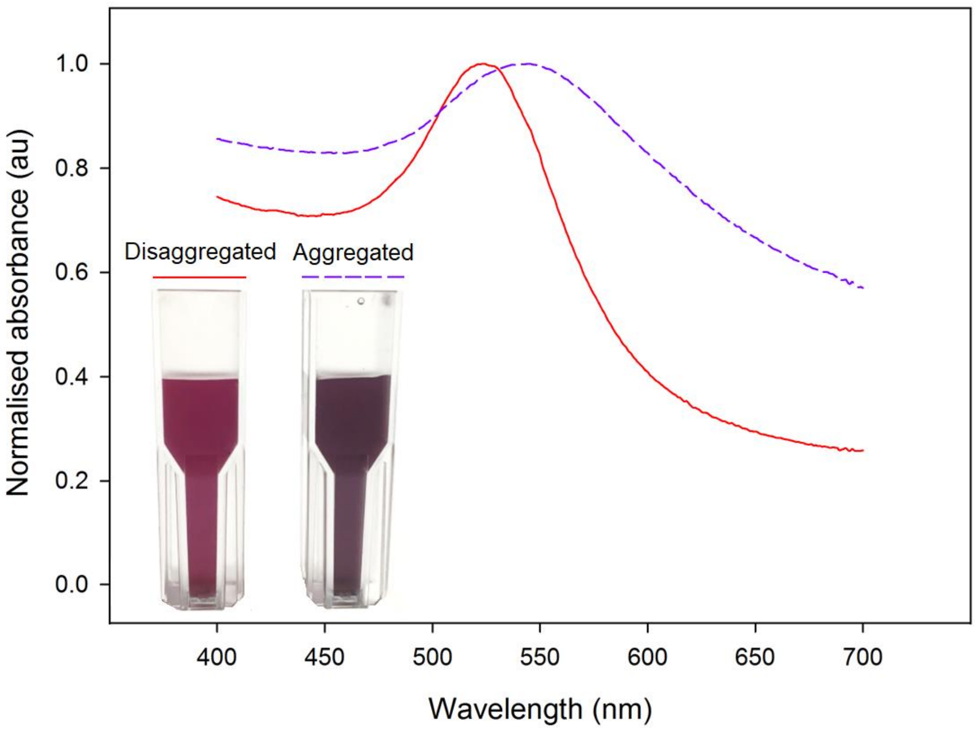

Figure S4. Absorbance spectra for disaggregated and aggregated gold nanoparticles. Absorbance maxima for free and aggregated nanoparticles were 524 nm and 544 nm, respectively. Inset image of disaggregated (*left*) and aggregated (*right*) gold nanoparticle suspensions.

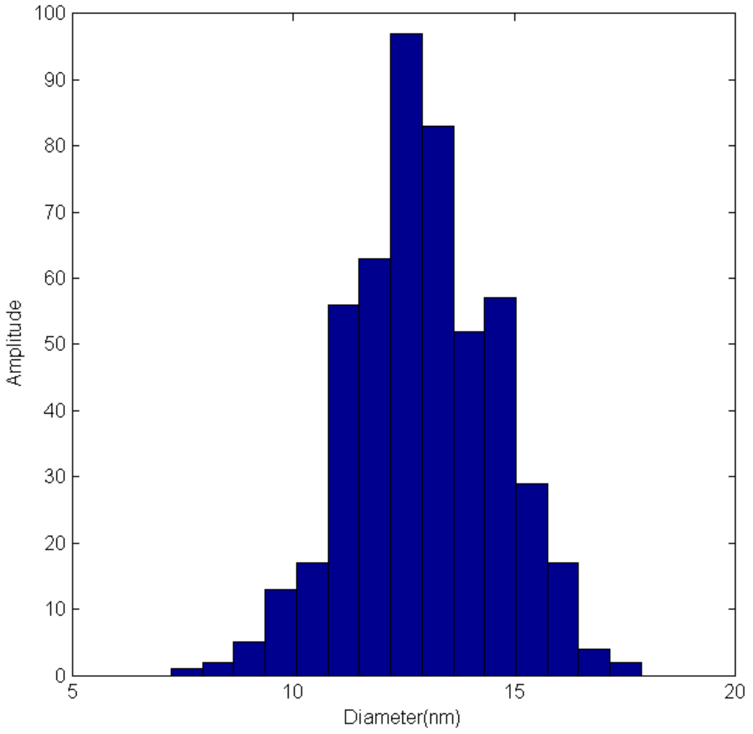

Figure S5. Histogram of unfunctionalized gold nanoparticle size distribution determined from TEM (n=50).

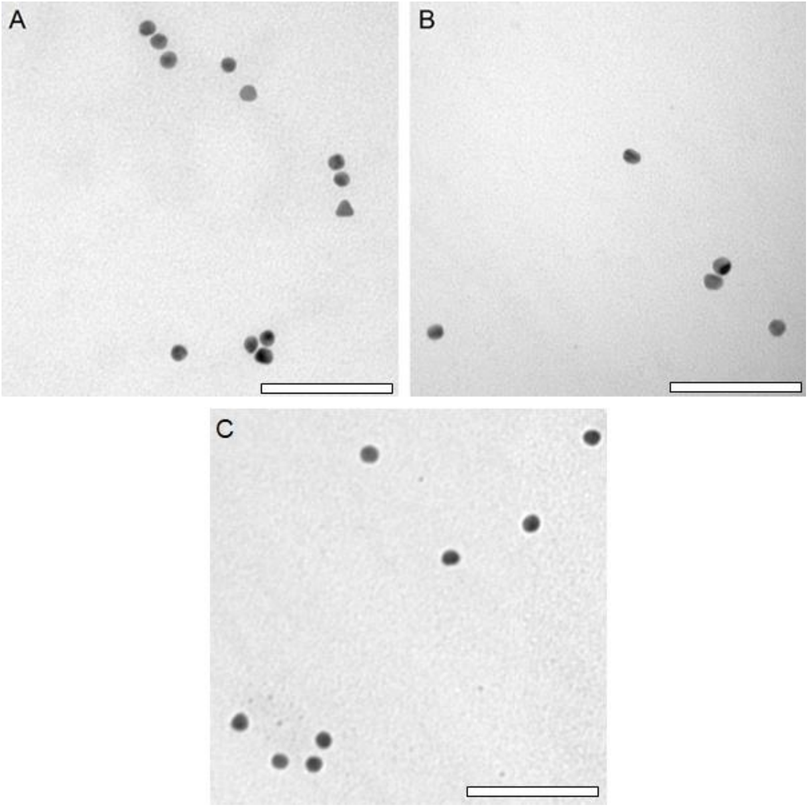

Figure S6. TEM images for gold nanoparticles functionalised with (A) *Sequence-A*, (B) *Sequence-B* and (C) after signal transduction pathway has been initiated following addition of conserved target enteroviral nucleic acid sequence. Scale bars = 100 nm.

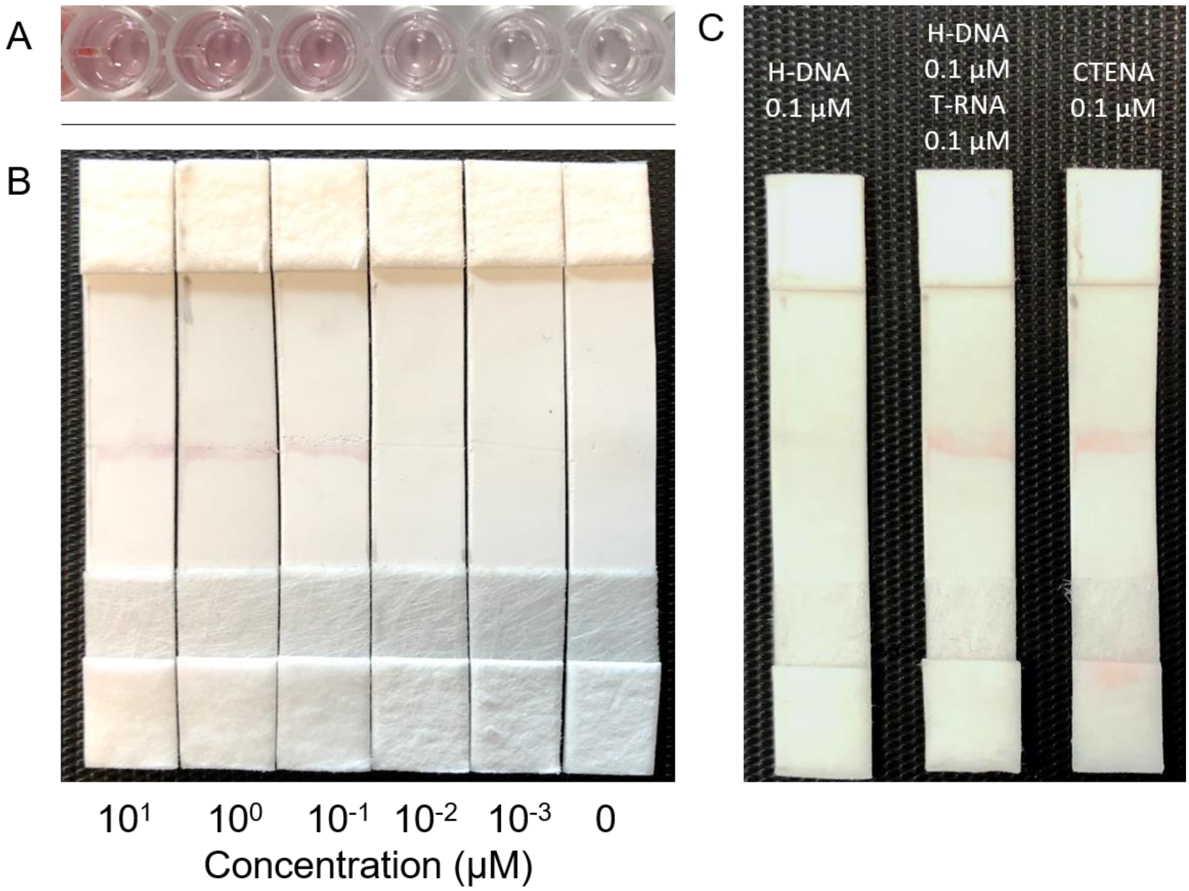

Figure S7. Response of gold-oligonucleotide-nanoconstructs to target viral DNA in the presence of human genomic DNA (0.1 µM), where (A) colorimetric change and (B) lateral flow assay. (C) Summary of lateral flow findings showing signal transduction mechanism of gold-oligonucleotide-nanoconstructs is not initiated in the presence of human genomic DNA (H-DNA, 0.1 µM) and does not affect triggering of signal transduction cascade in for conserved target enteroviral nucleic acid (CTENA, 0.1 µM).

SUPPORTING CAPTIONS

Supporting Movie 1. Rapid colorimetric response of gold-oligonucleotide-nanoconstructs in suspension when challenged with conserved target enteroviral nucleic acid (0.1 µM) and the binding of disaggregated biotin functionalised gold nanoparticles to streptavidin of lateral flow assays.

Supporting Movie 2. Differentiation of conserved target enteroviral nucleic acid positive and negative lateral flow cartridges using custom designed diagnostic.
